# Nursing Progress and Its Developments: VIII. Supporters of the College

**Published:** 1918-03-02

**Authors:** 


### THE MATRONS' AND SISTERS' DEPARTMENT.

### NURSING PROGRESS AND ITS DEVELOPMENTS.

### VIII.—Supporters of the College.

It has always been recognised that the future of any central nursing body must depend entirely on the support afforded to it by the heads of the leading training-schools in London, the provinces, and Scotland. The College of Nursing is not the first attempt to found a Council for the purpose of setting nursing affairs in order. It was initiated in the full knowledge of other failures to secure the adhesion of the main body of nurses to decisions affecting their welfare. It is important, however, to realise that it stands in a widely different position to-day from that attained at any time by other promulgators of nursing ideals. Former attempts failed because it was found impossible to secure the adhesion of the leading matrons actually responsible for the future of nursing. This time it is not a matter of proceeding from the outside to the inner fortress of nursing opinion, with the aim of securing perhaps one or two leading matrons to lend their sanction to a brave adventure. The College is itself the expression of the recognition by the leading matrons in the country, united as never before, that the hour has come for welding the nursing calling into a profession. It is the matrons themselves whose voice is heard in the call to nurses to rally round the College, and win for themselves and their sisters benefits greater than they guess.

There are three endowed hospitals in London with medical schools attached—St. Bartholomew's, St. Thomas's, and Guy's. Their matrons are respectively Miss A. McIntosh, R.R.C., Miss A. Lloyd Still, R.R.C., and Miss Hogg, who succeeded Miss L. V. Haughton, now unhappily retired from active work on account of ill-health. Miss McIntosh and Miss Lloyd Still are on the Council of the College, and Miss Haughton has but recently retired. Miss Swift, former matron of Guy's, where her work can never be forgotten, and Matron-in-Chief of the Joint War Committee, has also lent her support to the Council of the College, and we note with pleasure that the Navy is represented in the person of Miss Keenan, R.R.C., Head Sister Q.A.R.N.N.S., Royal Naval Hospital, Chatham. The Council numbers, too, among its most recent accessions of influence the name of Miss Sidney Browne, R.R.C., Matron-in-Chief of the Territorial Force Nursing Service, who has recently accepted the office of co-Honorary Treasurer, so that nurses have now one of their own body to look after their financial interests in the College. Of voluntary hospitals with medical schools attached, Miss Ray, R.R.C., matron of King's College Hospital, the largest voluntary hospital in London after the London, and Miss Cox-Davies, R.R.C., matron of the Royal Free, are both on the Council. The matrons of three Poor-Law infirmaries, Chelsea, West Ham, and Whitechapel, noted for their progressive nursing policy, are also members of the Council—viz., Miss Barton, R.R.C., Miss I. Clark, and Miss Mowat.

In the provinces, Miss Baillie, R.R.C., matron of the Royal Infirmary, Bristol; Miss E. M. Cummins, matron of the Royal Infirmary, Liverpool; Miss Musson, R.R.C., matron of the General Hospital, Birmingham; Miss Sparshott, R.R.C., lady superintendent of the Royal Infirmary, Manchester; and Miss Vincent, R.R.C., lady superintendent of the Royal Infirmary, Leicester, are active supporters of the College and on its Council, which also numbers one of the leading pioneers of provincial Poor-Law nursing in Miss Gibson, late matron of the Infirmary, Birmingham.

**In Scotland** the names of Miss Gill, R.R.C., lady superintendent of the Royal Infirmary, Edinburgh, and of Miss Melrose, R.R.C., matron of the Royal Infirmary, Glasgow, testify to the influential character of the support accorded to the Council by Scottish nurses. **In Ireland** the College has a young but enterprising branch, which is supported by Miss Curtin, matron of the Mater Infirmorum Hospital, Belfast, and by Miss Eddison, R.R.C., matron of the City of Dublin Hospital, both members of the Council. Finally, the names of Miss Hughes, late general superintendent of Queen Victoria's Jubilee Nurses; Miss Graham, of the Scottish Nurses' Association; and Miss Reed, Ivanhoe Nursing Home, Dublin, hold individually great authority in their respective fields.

